# A mass-customizable dermal patch with discrete colorimetric indicators for personalized sweat rate quantification

**DOI:** 10.1038/s41378-019-0067-0

**Published:** 2019-06-17

**Authors:** Vaibhav Jain, Manuel Ochoa, Hongjie Jiang, Rahim Rahimi, Babak Ziaie

**Affiliations:** 1 School of Mechanical Engineering, Purdue University, West Lafayette, IN 47907 USA; 2 School of Electrical and Computer Engineering, Purdue University, West Lafayette, IN 47907 USA; 3 Birck Nanotechnology Center, Purdue University, West Lafayette, IN 47907 USA

**Keywords:** Materials science, Engineering

## Abstract

In this paper, we present a disposable, colorimetric, user-friendly and mass-customizable dermal patch for chronological collection and discrete real-time in situ measurement of sweat secretion over a small area of skin. The patch consists of a laminated filter paper patterned into radially arranged channels/fingers with water-activated dyes at their tips. As channels are filled during perspiration, their tips change color once fully saturated, providing easily identifiable levels of water loss which in turn can be mapped to personal dehydration levels. The patch can be manufactured at low cost in a variety of sizes to allow hydration monitoring for individuals participating in activities under different conditions (intensity, temperature, humidity, etc.). Furthermore, we describe an analytical model that enables mass customization of such a flexible wearable system accommodating a broad range of sweat rates and volumes to generate patch designs that are personalized to an individual’s sweat rate, desired time of usage, and the temporal resolution of the required feedback. As a proof-of-concept demonstration, we characterized laser-fabricated patches that cover (7 cm × 5 cm) area of skin having various wicking materials, thicknesses (180–540 µm), and pore sizes (3–11 µm). Tests were conducted at various flow rates simulating different sweating intensities in the range of 1.5–15 mg/cm^2^/min. Experimental results for the case of a half-marathon runner targeting 90 min of usage and sweating at a rate of 1.5 mg/cm^2^/min indicated measurement accuracy of 98.3% when the patch is completely filled.

## Health: Showing the true color of dehydration

Scientists have developed a low-cost wearable skin patch that can monitor the level of perspiration from the human body, and could be used to track hydration levels in athletes, firefighters, and construction workers. Hydration is an important physiological marker, where even small deviations from normal levels can have negative impacts on a human’s cognitive and physical performance and can lead to serious conditions, such as heat exhaustion and stroke. Now, Babak Ziaie, Vaibhav Jain, Manuel Ochoa, and colleagues from Purdue University in the United States have developed a patch made from laminated filter paper patterned with “finger”-shaped channels that contain activated dyes which change color as perspiration increases, providing an inexpensive and adaptable way to monitor hydration levels. The patches can be mass customized into forms that are personalized to an individual.

## Introduction

Hydration is a delicate physiological parameter with even small deviations of 2% from normal levels negatively affecting a person’s cognitive and physical performance by over 30%^1^. Moreover, chronic dehydration can cause cramping and faintness, which can lead to more serious conditions such as heat exhaustion and stroke^2^. Hydration-related issues are especially common during endurance races, due to the inability of athletes to adapt their hydration needs to various factors and optimization of hydration by feel (i.e., subjective assessment) alone^3^. Studies have shown that more than half of all marathon runners have experienced a major decrease in running performance due to being dehydrated, and at least one-third of all marathon runners have suffered heat-related illness symptoms (e.g., severe muscle or stomach cramping, light-headedness, dizziness, nausea, or loss of ability to think clearly) during a run due to being dehydrated^4^. In these kinds of sporting activities, the most common cause of dehydration is sweat loss required for thermoregulation^5^. In some severe cases, this dehydration may even result in death if the fluid loss exceeds about 15% of the body weight and is not replenished^6^. In addition to dehydration, improper rehydration protocols can also lead to potentially fatal hyponatremia conditions^7^. Therefore, monitoring of hydration and sweat loss are very important during physically strenuous activities such as in sports; in addition to avoiding medical emergencies, it can also provide information about the athlete’s physiological capacity and efficiency under stress. These data can be used for preparing personalized training regimens and recovery schedules aiming toward better health and performance.

Despite such severe health effects, more than 70% of marathon runners don’t use any method to monitor their hydration status^4^. One reason for this is that measuring hydration is a nontrivial task requiring quantification of input−output water balance. Standard clinical and athletic approaches rely on the analysis of biomarkers such as blood serum ion concentration, urine color/osmolality, and body mass^8^. Each of these methods, however, poses various shortcomings such as the need for a nonportable scale to measure the athlete’s weight, delayed indication of dehydration in addition to difficult sample access and preparation in urine color, and invasiveness during blood serum sampling^9^. A more convenient approach is to directly monitor perspiration kinetics as a measure of hydration. Sweat-rate measurements have been conducted using a variety of techniques and devices, with the preferred standard for athletic field use being gravimetry for whole-body sweat rate analysis^10^. More conveniently, localized sweat rates are derived from the mass of sweat collected, or from changes in mass of the filter papers, patches, plastic pouches and ducts; but these methods are suited primarily to steady-state conditions and can sometimes result in a progressive occlusion of sweat ducts and sweat suppression^11^. They are also not real time and often require off-board analysis^10^. Several devices based on similar techniques such as Megaduct, Macroduct and Pharmchek have been commercially available since 1990 ^12^. The water vapor content of a gas can also be measured using a range of methods to quantify trans-epidermal water loss using various methods. The modern hygrometric technique of choice, where both timing and quantification precision is required, rely upon the effect of water vapor on electrical resistance and capacitance^10^. However, these methods can be slow and bulky, and they can lead to overestimation of sweat rates. Moreover, the trans-epidermal loss is only a small part of total cutaneous loss of water that needs to be replaced for optimum hydration.

Recently, researchers have developed more practical, and real-time sweat sensing patches with various sampling and sensing modalities that focus on rapid clinical assessments of pH^13–15^, temperature^16,17^ and various kind of sweat constituents/analytes^12,18,19^ rather than specifically focusing on massively growing hydration-conscious population^20^ for improving commercial/consumer adoption of sweat sensing. Often, they require multiple optical^21,22^, ampereometric^23,24^, electrochemical^25,26^ or bio impedance-based sensors^27–29^ and their measurements are nonlinear with respect to sweat rate due to ion reabsorption phenomenon^30^. Approaches such as these are suitable for clinical analyses where accurate quantification of dissolved analytes is more important than rapid assessment of perspiration rate and quantification for hydration-related information. Because most of these devices target detailed analysis of sweat based on presence of different analytes and are not specifically for sweat rate analysis, they are over designed which renders the system too expensive, bulky, non-real time, complex, unreliable, or nonportable, particularly for applications such as sports. Similarly, some sensors aim to be more accurate by detecting sweat rate using image analysis of commercial sweat collectors^31^; however, these systems are not real time and require post-processing.

For hydration monitoring in strenuous activities such as sports, construction environment, emergency management activities such as firefighting or military applications, low-cost patches with rapid real-time indication and simple readout methods would be more appropriate. Toward these, various kinds of direct flow rate sensors have recently emerged for application on skin. They include thermal/calorimetric sensors^32,33^, textile-based flow sensors^34^, PDMS-based flow sensors^35,36^ and paper-based flow sensors (paper is merely used as a passive pump for guiding sweat to some other sensor). These focus more on sweat rate measurements, but either are not real time, are bulky, or do not feature sufficiently high temporal resolution or range (or a combination of those) which keeps them out of reach for the majority of the global population for day-to-day usage and makes them difficult to scale up. Moreover, many of them require the wearer to interpret the results through complex steps, potentially diverting focus from other more important tasks (e.g., winning a marathon). Some of the latest PDMS-based sensors are driven by the eccrine pressure which can affect the sweat rate itself due to saturation. One of the biggest challenges in current devices is the personalized nature of sweat rate i.e., the inter-subject variability leading to no standard solution for all users^10,12^.

Athletes could benefit from a radically low-cost device that offers a more quantized feedback with an emphasis on chronological quantification of perspiration loss for fluid replacement without affecting the user body and can be customized to be used by any individual at mass production scale. None of the sensors and devices developed so far can be customized to individual user at scale.

To address the need for real-time user-friendly feedback for perspiration loss with chronological fluid replacement recommendations, while maintaining patch simplicity for scale-up at substantially lower costs (<$1 per device), we have developed a wearable paper-based platform that is mass-customizable with passive visual readout. The patch consists of a wicking material (paper) laminated between two polymeric films. The paper is patterned with a unique radial finger design that can offer linear discretized visual readout. We have also developed an analytical model that allows the patch to be mass customized in order to accommodate a broad range of sweat rates and volumes.

### Design overview

**Fig. 1:**
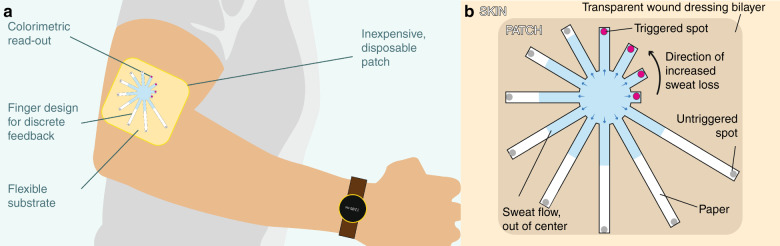
Device concept. **a** A wearable device/patch for measuring sweat rate and hydration status. **b** Conceptual illustration of the hydration monitoring patch with discretized levels of collected sweat which change color when fully wet for easy visibility

Efficient and accurate measurement of perspiration requires occlusion of a specific dermal region and collection of all the sweat secreted from that area for the duration of the measurement. A hydrophobic patch with an adhesive perimeter and an embedded hollow channel/tube can provide one such structure for sweat collection; however, sweat droplets forming at the skin surface may require hours to generate sufficient sweat for wicking into the channel for reliable readout. Instead, if the channel is filled with a hygroscopic material, any sweat secreted onto the skin can be immediately wicked and collected in the hygroscopic material for readout or transport. In this work, we present one such device that uses cellulose fiber as the wicking material. Rather than using a single channel, however, the design features a radial array of channels of varying lengths and widths that allow for controlled flow rates and quantization of the collected sweat for more control on objective and quantitative information. The radial design of the patch is illustrated in Fig. 1a, b. Such a design having discretized “fingers” in increasing order of length can allow chronological tracking of fluid because of design-controlled (uniform in case of equal width) flow rate in each finger achieved by the circular pattern. This design also allows easy loading of water-activated dyes, such as CoCl_2_, which is blue in its anhydrous state and turns red when hydrated (${\mathrm{CoCl}}_{\mathrm{2}}\cdot {\mathrm{6H}}_{\mathrm{2}}{\mathrm{O}}$), at the tip of the fingers to provide visual cues to the user. This substrate is sandwiched between two films of a waterproof material having a port on one side from which the fluid (i.e., sweat) can enter the substrate. To maintain the uniformity of flow in the fingers, the port should be concentric with the central circular portion of the substrate, and its diameter should be less than or equal to the central circular portion of the substrate.

### Patch usage

The sweat patch aims to help people maintain constant hydration status, which can otherwise vary as a result of change in exercise intensity, indoor/outdoor climate, or personal factors (e.g., stress level). The design presented here allows personalized (for professional athletes) and generic versions (for the general public) to be created with ease.

Lay people who want to gauge their water loss throughout the day or while they exercise can do so using a mass-produced, off-the-shelf patch with design catering to the perspiration rate of the average person. To use it, the person would don it either at the beginning of the day or at the start of their exercise routine. As the person sweats during the day or while exercising, the patch will gradually fill up, with each finger of the patch indicating a specific volume of secreted sweat. At the end of the day or exercise session, the person can check the patch to see how many fingers filled up; with the help of a chart (or in a future implementation, with the same information relayed via a smartphone app), they can determine how much water they lost to perspiration so that they can be sure to replenish it.

For professional athletes, the patch can be further customized to the perspiration rate of the individual and furthermore to their specific activities. This capability is important because the patch has an upper limit (tunable via design) for the amount of sweat that it can monitor; thus, a patch that fills up in 1 h of moderate exercise by an average person may fill up within minutes for a professional athlete involved in high-intensity training. To withstand an hour for such cases, the athlete can order custom patch designs. To do so, the athlete would first wear a set of calibration patches (of different capacities, including one guaranteed not to fill up with hours of extreme exercise) and submit a photograph of them through some means such as via a smartphone app after their routine along with the routine duration. The patch manufacturer would then use the photographs to create a custom patch that would fill up to an exact marked point (e.g., 80% of the patch fingers) for that routine for that specific person, which the athlete can then purchase in bulk. When the athlete wears those patches for the indicated activity in subsequent sessions, he/she can use them to precisely gauge hydration consistency for that routine by checking the sweat level at the end of the routine; if the patch does not fill to the expected finger, a companion instructions chart (or app, in the future) can let them know how much water difference this corresponds to (based on the specific geometry of their patch) so that they can adjust their water intake or exercise intensity accordingly.

Since the patch design is rationally customizable, it is feasible to manufacture custom patches for multiple routines for each power user. Thus, the customizability of this patch design can allow both high-intensity athletes as well as the general public to introduce hydration consistency into their work or exercise routine even in varying climates, i.e. a custom patch can be created. The following subsection provides an analytical model for determining the geometric parameters of the patch based on the target use specifications.

### Analytical model

**Fig. 2:**
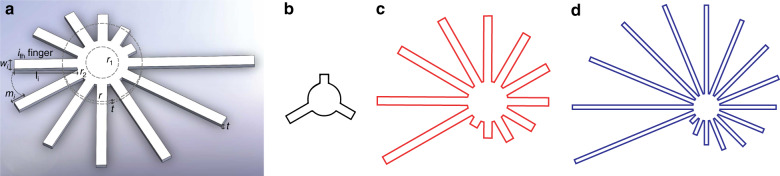
Patch parameters and examples of customization. **a** Schematic of the patch showing various geometrical design parameters. Comparative geometric designs for three use cases: **b** office work, **c** half marathon, and **d** ultramarathon

Figure 2a is a schematic of the patch showing various geometrical design parameters. Assuming *Q*_s_ to be the sweat rate per unit area (mg/cm^2^ min) of the skin, *Q* the wicking flow rate (mg/min) in the strip*, N* the number of strips on a patch, *t* the thickness (cm) of the wicking material, *w* the strip width (cm), and *l*_*n*_ the length of the *n*th strip (cm, assuming all strips have the same width and thickness). If the first strip is 2 mm long and each subsequent strip is 2 mm longer, then $l_n = nl_1$.

Let the time required to fill the inner circle be *T*_0_ and the time required to fill strip *n* be *T*_*n*_. Then, the time to fill a patch is given by

1 $$T = T_0 + \mathop {\sum }\limits_{n = 1}^N T_n = T_0 + \mathop {\sum }\limits_{n = 1}^N \frac{{ntwl_1}}{Q} = T_0 + \frac{{twl_1}}{Q}\mathop {\sum }\limits_{n = 1}^N n = T_0 + \frac{{twl_1N(N + 1)}}{{2Q}},$$

Let $A = \pi r^2$ be the area of the central port (in cm^2^) and ${\mathrm{\Phi }} = AQ_{\mathrm {s}}$ the incoming sweat flow rate (mg/min) in the device from skin. Now because of the radial design, if $Q = {\mathrm{\Phi }}/N$ (the flow rate is divided equally among the strips), then the total time to fill the patch is as follows.

2 $$T = T_0 + \frac{{twl_1N^2(N + 1)}}{{2{\mathrm{\Phi }}}}.$$

The temporal resolution (in minutes), defined as the time in between patch readings (e.g., minutes for running cases, hours for sedentary situations), can be written as follows.

3 $$R = \frac{{T - T_0}}{N} = \frac{{twl_1N(N + 1)}}{{2Q_{\mathrm {s}}A}}.$$

Rearranging (3):

4 $$\frac{{wt}}{A} = \frac{{2RQ_{\mathrm {s}}}}{{l_1N(N + 1)}}.$$

Assuming $T_0 \ll T$, we can say $N \approx T/R.$

(Note: $T_0 = V/Q_{\mathrm {s}}A = tA/AQ_{\mathrm {s}} = t/Q_{\mathrm {s}},$ where *V* is the volume of the central circular region. Hence *T*_0_ is negligible, when *t* is small and *Q*_s_ is high. This determines accuracy of first-order model $N = T/R$.) Combining these together results in

5 $$\frac{{wt}}{A} = k,$$

where *k* is a constant for a given set of user conditions (duration of the activity while wearing the patch, desired temporal resolution, and sweat flux). Specifically,

6 $$k = \frac{{2R^3Q_{\mathrm {s}}}}{{l_1T(T + R)}}.$$

In addition, theoretically, the geometry must obey the following condition, $Nw\, < \,2\pi r$, which limits the resolution. The *t* is practically restricted to the thickness of commercially available papers. Moreover, since human eccrine sweat glands are typically distributed with a density of 16–532 glands/cm^2^ (up to 700 glands/cm^2^), solutions should be limited to conditions where $A\, >\, 6.25\,{\mathrm{mm}}^2$ ($r\, >\, 1.4\,{\mathrm{mm}}$) to ensure at least one sweat gland is covered by the port even in regions with sparse gland coverage^10,37^. The minimum width *w* possible is governed by the resolution of the manufacturing technique (30–50 µm for the CO_2_ laser used in our laboratory, depending on the material used).

Together, the above equations define the design parameters for the sweat collection patch and enable mass customization of patches based on the required usage conditions. The desired profile of the feedback can be controlled by controlling thickness, length and width of individual strips. Other improvements can also be made to the design for increasing the accuracy of the first-order model by removing the central region completely as an alternate design and having just a central ring.

### Parameter values for example uses cases

To determine the practicality of this design methodology (i.e., whether resulting designs are manufacturable and practical for wearing), we calculated the patch geometry required for using the patch in three different use cases: an office person in a non-air-conditioned environment using the patch for 30 min on the upper arm (with feedback at every 10 min), a competitive half marathon runner with patch on the dorsal hand targeting less than 90 min time to complete the race or ~6.5 min per mile pace, and a person exercising for 8 h (such as in an ultramarathon) using the patch on his chest (with feedback after every 30 min). Here, the sweat rate values for all the three cases have been taken from Taylor et al.^10^. The parameter values are shown in Table 1 along with the resulting patch designs obtained using the analytical model as shown in Fig. 2b–d; these specific designs are practical (manufacturable and wearable), suggesting that the model can be used for low- as well as high-perspiration use cases.

**Table 1:** Geometric and other relevant parameter values for three use cases. Sweat rate values were obtained from ref. ^10^

Design parameter	Office work	Half-marathon*	Ultramarathon
Time of application, *T* (min)	30	90	480
Sweat rate, *Q*_s_ $\left( {\frac{{{{\mathrm {mg}}}}}{{{{\mathrm {cm}}}^2 \times {{\mathrm {min}}}}}} \right)$	0.25	1.53	0.90
Paper thickness, *t* (µm)	50	180	500
*r* (mm)	5	11	10
Radius of the input port, *r*_1_ (mm)	4	4	3.4
Temporal Resolution, *R* (min)	10	6.5	30
Number of fingers, *n*	3	12	16
Gap b/w fingers, *m*_*i*_ (mm)	2	2	2
Width of the fingers, *w* (mm)	2	2	1
Density of sweat $\left( {\frac{{{\mathrm {mg}}}}{{{\mathrm {cm}}^3}}} \right)$	1000	1000	1000

^*^Used for representative experiments in this work

## Results

### Qualitative inspection and mechanical assessment

**Fig. 3:**
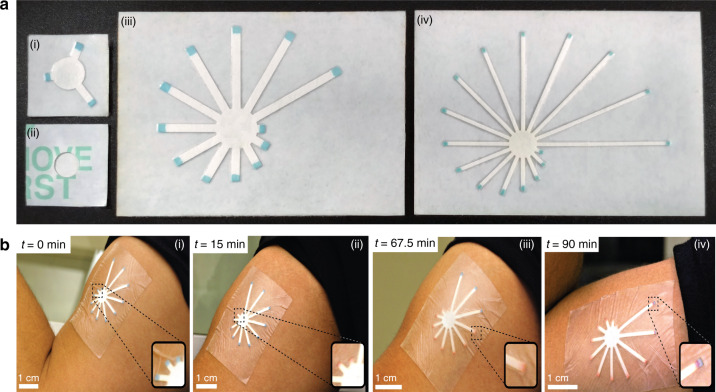
Prototypes and usage. **a** Fabricated prototypes of each of the three designs corresponding to the three use cases (i) office person (front side), (ii) office person (back), (iii) half marathon runner, and (iv) ultramarathon runner. **b** The sweat patch being used on the upper arm of a person working out in the gym showing the sequentially changing color of the tips from blue to red as a function of exercise time (i)−(iv)

Prototypes of the patches corresponding to the three use cases of the office resting person, half marathon runner, and the ultramarathon runner were fabricated and are shown in Fig. 3a (i−iv). The second use case prototype of the sweat patch is shown being used by an athlete during an aerobic workout in Fig. 3b (i−iv). The patch can flex sufficiently to conform to the athlete’s skin, and it remains attached to the skin throughout the workout. The patch readout is visible in standard gymnasium lighting. These qualitative investigations demonstrate that the patch is a practical wearable device with sufficient robustness for active wear. The results of the peel test are shown in the Supplementary Material and reveal a bond strength in the range of 2–8 N for a peel distance of 5 cm (equivalent to 103–412 g/in), a range comparable to that of commercial bandages^38^.

### Comparison of hygroscopic materials

**Fig. 4:**
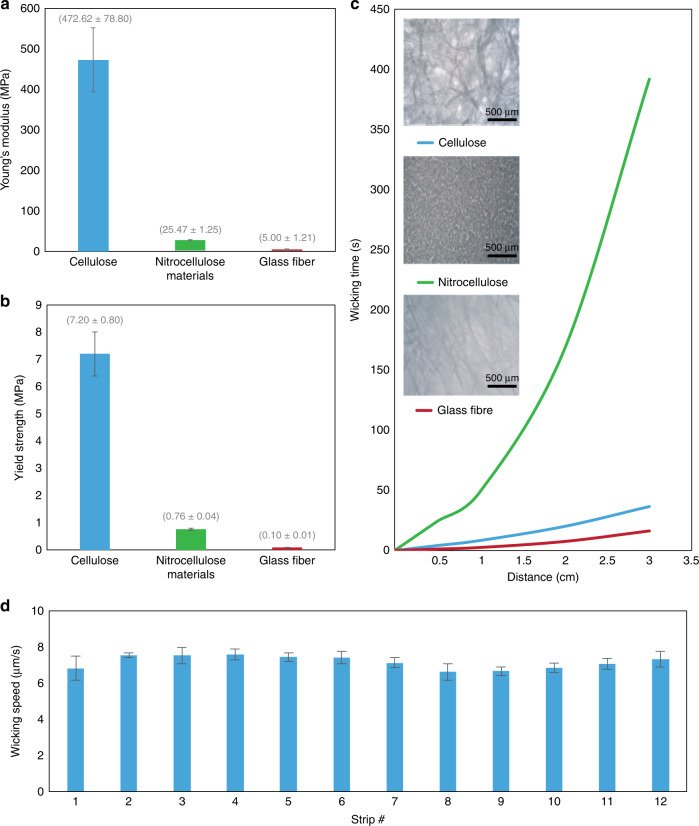
Material properties and selection. **a** Elasticity (Young’s modulus) of the three wicking materials. **b** Yield strength of the three wicking materials. **c** Wicking time for different materials (strips of width 2 mm and length as mentioned in experimental section) (optical microstructures are shown in insets). **d** Average wicking speed of the fluid in each strip under 0.05 mL/h flow-limited wicking of sweat in the patch (simulation of real scenario); the wicking speed for each strip varies by no more than 9.7% and the mean wicking speed among all strips is consistent within 4.96%

The results of the mechanical testing are summarized in Fig. 4. Paper is the strongest and most ductile among the three tested materials, having an average Young’s modulus of 472 MPa and yield strength of 7200 kPa (Fig. 4a, b). The average wicking speed of each of the three materials cellulose, nitrocellulose and glass fiber was found to be 3 × 10^−4^, 8 × 10^−5^ and 9 × 10^−4^ m/s, respectively (Fig. 4c). Paper exhibited a wicking speed higher than nitrocellulose, but lower than glass fiber. The wicking speeds for various strips are shown in Fig. 4d.

### Patch performance

**Fig. 5:**
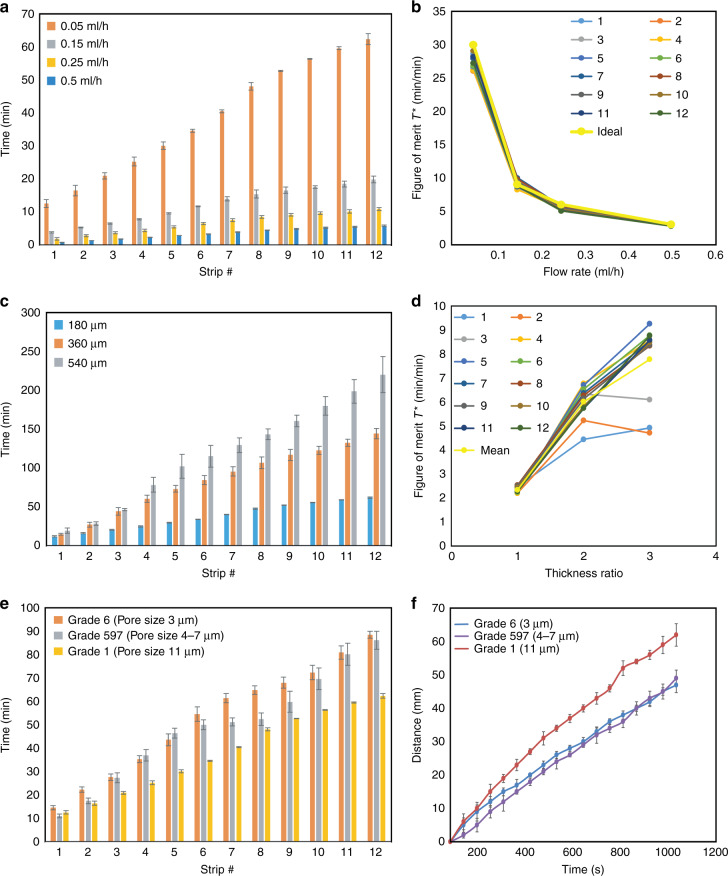
Patch performance as a function of design parameters. **a** Time taken by the strips to fill as a function of flow (sweat) rate. **b** Figure of merit T* as a function of flow rate. **c** Time taken by the strips to fill as a function of device thickness. **d** Figure of merit T* as a function of device thickness ratio. **e** Time taken by strips to be filled as a function of material pore size. **f** Distance traveled by the fluid (therefore, the wicking speed) under flow-limited imbibition at the flow rate of 0.05 mL/h as a function of pore size. All experiments (*n* = 5)

The device performance is a function of the person’s sweat rate (*Q*_s_), thickness (*t*) of the wicking layer, and material properties of the wicking layer (e.g., pore size distribution). The results of the experimental performance tests of the patches under flow-limited wicking are shown in Fig. 5; such condition adequately simulates real use cases since physiological and environmental limitations of perspiration rate manage the flow rate of sweat into the patch (as long as the wicking rate of the material is higher than the sweating rate). The performance of the device under different conditions is discussed further in the Discussion section. The patches characterized here were designed per the specifications of the half-marathon runner use case (described above, with the patch used on the dorsal region of the hand).

## Discussion

The developed patch quantitatively tells the user about the amount of sweat lost as a function of the filled strip number (identified by color change on the end of the tips). For example, in Case 2 (the patch designed for the half marathon user and used for experimental validation in this paper), the central circular region corresponds to 324 mL of total body sweat loss; the second strip being filled (Fig. 4b (ii)) corresponds to 401 mL of sweat loss; the ninth strip (Fig. 4b (iii)) corresponds to 1.484 L of sweat loss; and the filling of the entire patch (Fig. 4b (iv)) corresponds to 2.33 L of whole-body sweat loss. The use of a commercial medical film as the primary layers helps to promote its translation to market. Moreover, the low moisture vapor transmission rate (MVTR) of this particular film is 249−443 g/(m^2^ 24 h) or about 0.02−0.03 mg/(cm^2^ min), which is significantly smaller than the rate of sweat production considered in Table 1 (i.e., 0.2−1.53 mg/(cm^2^/min))^37^. Thus, evaporation effects should be negligible and most sweat should be captured in the wicking material. Although occlusive dressings can increase moisture accumulation on the skin and may lead to progressive blocking of sweat ducts and sweat suppression^11,39^, the use of highly absorbent filter paper material with a design that is customizable to various sizes can help to prevent sweat build-up by wicking it into the patch as it is produced^10,40^.

### Comparison of hygroscopic materials

A suitable material for use as a wicking material in a sweat collection platform must be economical, flexible, strong, not brittle and hygroscopic. We compared three common wicking materials in terms of their mechanical properties, cost, and wicking characteristics. Each of the three investigated materials has several advantages and disadvantages (Fig. 4). Paper is inexpensive ($0.05 per patch) and is the strongest and most ductile among the three tested materials, having an average Young’s modulus of 472 MPa and yield strength of 7200 kPa (Fig. 4a, b). However, its loose fibrous nature limits the types of dyes which can be sharply immobilized. Furthermore, its pore locations and sizes are not strictly controlled (as opposed to nitrocellulose which is an artificial membrane with precisely etched holes providing a more uniform morphology and superior dye retention properties).

Nitrocellulose has the lowest absorption rate (wicking speed) among the three materials. However, it is expensive ($1.50 per patch), weak, and brittle (Young’s modulus of 25.4 MPa and yield strength of 760 kPa), making it difficult to handle during patch fabrication and during use. Glass fiber filter paper has a higher absorption capacity (wicking speed) than the other two materials; however, it is also the most expensive of the three ($3 per patch), has low tensile strength (Young’s modulus of 5 MPa and yield strength of 96 kPa) and is difficult to immobilize dyes since it consists of a collection of loosely assembled glass fibers. With these design tradeoffs, we determined cellulose paper to be an adequate choice for the sweat wicking patch.

### Performance as a function of flow rate

Since practical use will expose the patch to many different flow rates (sweating intensities), it is important to investigate the effect of the flow rate on the performance of the sweat sensor. The effect of flow rate was characterized using strips of various lengths and materials. As shown in Fig. 5a, the time taken for sweat to reach the end of each strip as a function of strip length (and strip number) is linear (*R*^2^ = 0.9928 for flow rate *q* = 0.05 mL/h, 0.9886 for *q* = 0.15 mL/h, 0.9895 for *q* = 0.25 mL/h, and 0.9926 for *q* = 0.5 mL/h). Such linearity is convenient, as it makes the entire patch a linear system, which allows for simple calculations (e.g., using a companion app) for demining perspiration volume, and it enables more data-driven users to predict (during their exercise activity) when the next strip will be filled and plan their hydration strategy appropriately.

To be able to compare the flow rate performance characteristics among strips of different lengths, it is useful to define a normalized parameter to be used as a figure of merit:

7 $$T^ \ast = \frac{{T - T_0}}{d},$$

where *T* is the time required to saturate a strip of length *d*, and *T*_0_ is the time needed to completely fill the initial central circular portion of the patch. The volumetric equation for each strip is,

8 $$\left( {T - T_0} \right)q^ \ast = Ad,$$

where $A = tw$ is the cross-sectional area of the strip, and $q^ \ast = q/N$ is the flow rate in the strip (i.e., total flow rate (*q*) at the inlet of the patch, divided by the number of strips *N*). Combining Eqs. 7 and 8 yields the normalized equation for any strip, $T^ \ast = Ntw/q$. Figure 5b depicts this theoretical characteristic curve for *N* = 12 strips, labeled as the “Ideal” yellow curve. The actual measured performance of individual strips (#1-12 for a 12-strip patch) are also plotted together with the yellow curve for comparison. The plot shows that the measured data differ from the theoretical curve by less than 14.5% for flow rate *q* = 0.05 mL/h, 7.6% for *q* = 0.15 mL/h, 14.4% for *q* = 0.25 mL/h, and 8.6% for *q* = 0.5 mL/h. Moreover, the various strips differ from each other by no more than 5.6% for flow rate *q* = 0.05 mL/h, 6.6% for *q* = 0.15 mL/h, 6.2% for *q* = 0.25 mL/h, and 4.2% for *q* = 0.5 mL/h, confirming that the theoretical model for a strip faithfully reproduces the flow kinetics of strips of various lengths in a patch.

### Performance with respect to thickness

It is also important to investigate the effect of substrate thickness on sensor performance, since extreme situations (i.e., very high or very low sweat rates) would require tuning of the thickness for optimal temporal range. Figure 5c and its normalized version Fig. 5d are the result of the investigations of device performance upon scaling of substrate thickness in Eq. 6 along with the figure of merit defined with respect to the time ($T^ \ast = (T - T_0)/d$, $t^ \ast = (t/180)$, $T^ \ast = 12\left( {\frac{w}{q}} \right)t^ \ast$ for each strip) at a fixed flow rate of *q* = 0.05 mL/h. The strip saturation time as a function of strip # increases linearly ($R^2 = 0.9889$ for *t* = 180 μm, $R^2 = 0.9815$ for 2*t* = 360 μm and $R^2 = 0.988$ for 3*t* = 540 μm) with increasing thickness, but its variability within the strips for a particular thickness also increases with thickness (standard deviation at $t^ \ast = 1$: 5.1%, at $t^ \ast = 2$: 11%, at $t^ \ast = 3$: 20%). This increase in variability is understandable since increasing thickness (and hence cross-sectional area) increases capillary forces, resulting in more flow variation in thicker materials. The data suggest that longer strips are needed when using thicker materials to ensure reliable modeling of the flow characteristics in the strips.

### Performance with respect to porosity

Figure 5e represents the performance of the device when cellulose filter papers of different pore sizes are used to make the device. The observed variability of the microfluidic flow with pore sizes can be qualitatively explained using a parameter termed hydraulic conductivity. This parameter describes the ease of fluid flow through pore spaces or fractures, and it depends on many factors, including the intrinsic permeability of the material, the degree of saturation, the density and viscosity of the fluid, the pore size, pore geometry, pore spatial distribution, and the environment conditions.

One way to make the material choice with respect to porosity is to characterize the materials in terms of their wicking speed by conducting linear wicking tests under similar flow-limited imbibition conditions at the flow rates of water simulating those of sweat from body. Figure 5f reveals the results of this experiment suggesting the variation of wicking speed (or alternatively time taken to transverse a fixed length) with respect to the cellulose paper pore size. The ratio of wicking speed of water in paper having 11 μm pores (Grade 1) and 3 μm pores (Grade 6) is found to be $T\left( {3\,{{\upmu {\mathrm {m}}}}} \right)/T\left( {11\,{\upmu\mathrm{m}}} \right) = v\left( {11\,{\upmu\mathrm{m}}} \right)/v\left( {3\,{\upmu\mathrm{m}}} \right) = 1.36$. This information obtained from characterization of materials can be used for device level calculations as the same ratio is also maintained in the device as seen in Fig. 5e. We see that the ratio of total time taken to fill the patches does not change, $T\left( {3\,{\upmu\mathrm{m}}} \right)/T\left( {11\,{\upmu\mathrm{m}}} \right) = 1.39 \sim v\left( {11\,{\upmu\mathrm{m}}} \right)/v\left( {3\,{\upmu\mathrm{m}}} \right) = 1.36$.

The Grade 6 paper equation becomes a line with $R^2 = 0.9866:T({\mathrm {min}}) = 6.58n + 10$, where *n* is the strip number. For this Grade 6 paper, the patch lasts for an average of 88.5 min with about 1.7% of error among trials; the result suggests that this patch would be suitable for use by a marathoner over a 90-min time period. The performance of the strips also is very close (<12% error for the first strip, and as low as 1.7% error for the 12th strip) to the theoretically designed/required $T = 6.5n + 12.5$. Hence, the results validate the design methodology in being effective to be used for designing customized patches according to the input parameters of person-specific sweat rate, required time of usage, size of the patch and desired resolution with few precautions to be taken in cases of extreme cases/limits including delaying the first point of feedback to a suitable value.

#### Conclusion

Overall, we have developed a patch for real-time in situ perspiration monitoring and quantification with a robust customizable design that can be tuned to any user (based on biometrics) or application. In cases where the dominant mode of hydration loss is through sweat, it can provide a first measure of dehydration and can be used to provide optimum fluid replacement strategies. The patch can be mass customized to handle a broad range of sweat rates and has been validated against flow rates of 0.05, 0.15, 0.25 and 0.5 mL/h (equivalent to sweating intensities in the range of 1.5–15.3 mg/(cm^2^/min)) wicking material thicknesses of (180, 360, and 540 μm), and material porosities of (3, 4–7, and 11 μm). Owing to the cost, strength, availability, wicking properties and dye retention capabilities, the substrate to be used for the case of half marathon (when the desired location of the patch is the back of the hand) has been chosen to be Grade 6 cellulose filter paper. A patch for the use case of a half marathon runner targeting 90 min of usage and sweating at a rate of 1.526 mg/(cm^2^/min) from the dorsal region of the hand was designed, fabricated, and experimentally tested for performance indicating an accuracy of the design within 1.7% of the desired parameters.

## Materials and methods

### Fabrication process

Numerous hygroscopic materials and hydrophobic films can be used as the active layer and encapsulating layers, respectively, of the patch. In this proof-of-concept demonstration, we selected a medical-grade polymeric wound dressing, Opsite (Smith and Nephew) transparent film (or a combination of polyurethane film and polymethacrylate based adhesive), as the encapsulating layer due to its commercial availability and established use in clinics. For the channel material, we investigated the hygroscopic, mechanical, and economical properties of three commercial filter papers commonly used for wicking-based transport of liquids in laboratory and industrial applications: cellulose acetate, glass fiber, and nitrocellulose. The experiments detailed in next section show that of these three, cellulosic filter paper (cellulose acetate) provides a reliable, mechanically robust, and economical solution for the present sweat sensor platform.

**Fig. 6:**
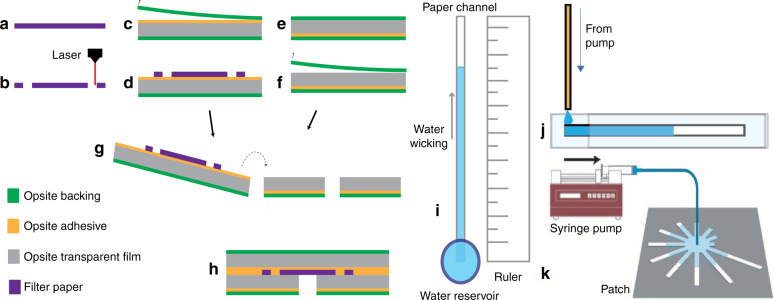
Patch fabrication process and characterization. **a**, **b** Laser machine filter paper, **c**, **d** expose adhesive of one polyurethane film and attach the paper, **e**, **f** Laser machine wicking port on another piece of film and remove the top backing, **g**, **h** stack together films for assembly. Remove backings before use. Test setup for material wicking speed characterization: **i** unlimited-source wicking, **j** flow-limited wicking. **k** Patch characterization setup using a syringe pump

The fabrication process of the patch is shown in Fig. 6a−i. First, the paper is laser-machined into the multichannel pattern using a laser engraver^41,42^. Next, a water-activated dye, anhydrous CoCl_2_, is deposited on the paper strip tips. This is done via drop casting of ${\mathrm{CoCl}}_2.6{\mathrm{H}}_2{\mathrm{O}}$ followed by a subsequent heat treatment to drive out the water. Although for this work, the dye was applied manually, this step can also be achieved in a more automated and scalable fashion through stamping or other printing techniques (e.g., inkjet printing, screen printing) by the development of stable, printable inks, as has been previously presented by our group^43^. One piece of medical-grade semipermeable waterproof Opsite film (or similar polyurethane-based films with methyl acrylate or comparable adhesive) is then laser-machined to create the inlet port. The three layers are then stacked such that the hole in the bottom layer film is concentric with the circle in the paper pattern. During use, the backings are removed, and the paper is exposed to the skin via the hole as indicated in Fig. 6h.

### Characterization of the hygroscopic materials

Three wicking materials were characterized in this work: cellulose paper, glass fiber paper, and nitrocellulose paper. The schematic of the setup is shown in Fig. 6i, j. For each material, strips of size 40 mm × 2 mm were cut with a laser engraver system to ensure uniform length and width among test samples. To characterize the wicking properties under unlimited-source conditions, a strip was placed alongside a ruler, with a plastic reservoir (1 mL volume, open on the top and perforated at the bottom) placed over one end of the strip to serve as the source of liquid for wicking (0.2 M aqueous solution of Evans blue dye); whereas, for the limited-source case, a fixed volume of the liquid was placed at one end of the strip. The wicking rate was calculated by measuring the time required for water to wick to various distances. Similarly, the wicking speeds under flow-limited conditions (forced imbibition) at controlled flow rates using a syringe pump were measured using a setup shown in Fig. 6j.

The mechanical integrity of the materials was evaluated in terms of their ultimate tensile strength and elastic modulus using a tensile testing machine (UTM, ADMET Inc, eXpert 4000). Failure modes of the patch were inspected by stretching the fabricated patch of use case 2 design as described in the theory section (of size *l*_1_ = 2 mm, *n* = 12, *t* = 180 µm, *r*_1_ = 8 mm, *r*_2_ = 1 cm, *w* = 2 mm and *m* = 2 mm). Samples of 2 cm length and 2 mm width were tested with the UTM under different strain rates. Initial experiments on the strength of papers in response to various strain rates of 10, 0.2 and 1 mm/min revealed negligible variations. Subsequent studies focused on evaluating the paper at a single strain rate, namely 10 mm/min. All substrate samples were inspected and imaged using Bausch & Lomb Micro Zoom 2 high performance microscope.

### Characterization of the sweat sensing patch

For characterization of the patch, sweat secretion from the skin into the patch was simulated by using syringe pump having a 3 mL BD syringe with needle of size BD 18G1½ attached to a tube of inner diameter 800 µm. This was further attached to a BD27G1/2 needle (diameter 0.4 mm) on the opposite end supported by a clamp stand. The flow was delivered perpendicular to the patch, at the center of the port of the patch. The patch from use case 2 (i.e., half marathon runner), of size, *l*_1_ = 2 mm, *n* = 12, *t* = 180 μm, *r*_1_ = 8 mm, *r*_2_ = 1 cm, *w* = 2 mm and *m* = 2 mm, was attached to the test platform using tape on four sides, and the centering was done using a ruler. Various flow rates simulating different sweating intensities (0.05, 0.15, 0.25 and 0.5 mL/h or (1.526–15.26) mg/cm^2^/min), patches with different thicknesses (180, 360, 540 μm), and with cellulose-based papers of different porosities (3, 4–7, 11 μm) were characterized. The time at which the sweat surrogate (water) reaches the tip of the fingers was recorded using an automated camera setup.

The adhesion strength of the patch to skin is an important parameter for any patch/bandage; therefore, we investigated the bond strength between the patch and skin via a 90° and a 180° peel test using a tensile testing machine (UTM, ADMET Inc, eXpert 4000). A 5 cm × 7 cm patch was attached to the forearm and subsequently peeled off using the tensile testing machine at a rate of 5 cm/min, while the applied load and displacement were recorded over time. The experiment was conducted at least in triplicate.

## Supplementary information

Supplementary Information
