# Supplementary material for: A mass-customizable dermal patch with discrete colorimetric indicators for personalized sweat rate quantification
Source: Microsyst Nanoeng. 2019 Jun 17;5:29. doi: 10.1038/s41378-019-0067-0 (PMC6572848; doi:10.1038/s41378-019-0067-0)
Supplement: Supplementary file 1 — Supplementary Information [file 41378_2019_67_MOESM1_ESM.pdf]

SUPPLEMENTARY INFORMATION

A Mass-Customizable Dermal Patch with Discrete Colorimetric Indicators for Personalized Sweat Rate Quantification

Vaibhav Jain,[abc] Manuel Ochoa,[bc] Hongjie Jiang,[bc] Rahim Rahimi,[bc] and Babak Ziaie[bc*]

a) School of Mechanical Engineering, Purdue University, 47907, USA
b) School of Electrical and Computer Engineering, Purdue University, 47907, USA
c) Birck Nanotechnology Center, Purdue University, 47907, USA

1. CONCEPT, DESIGN AND THEORY

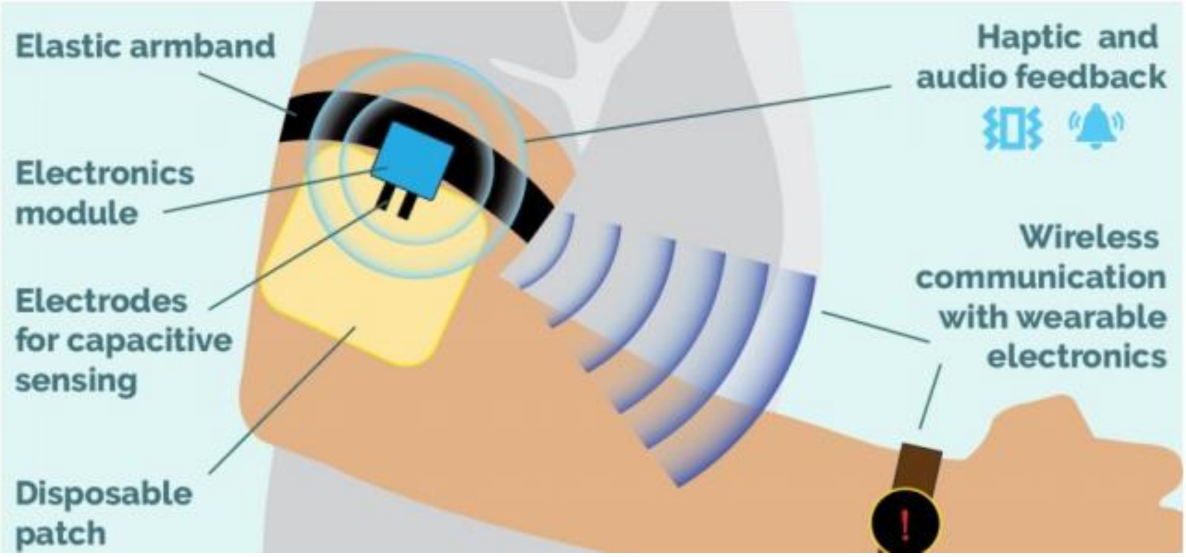

Figure S1: A wearable system consisting of a paper platform and a feedback module.

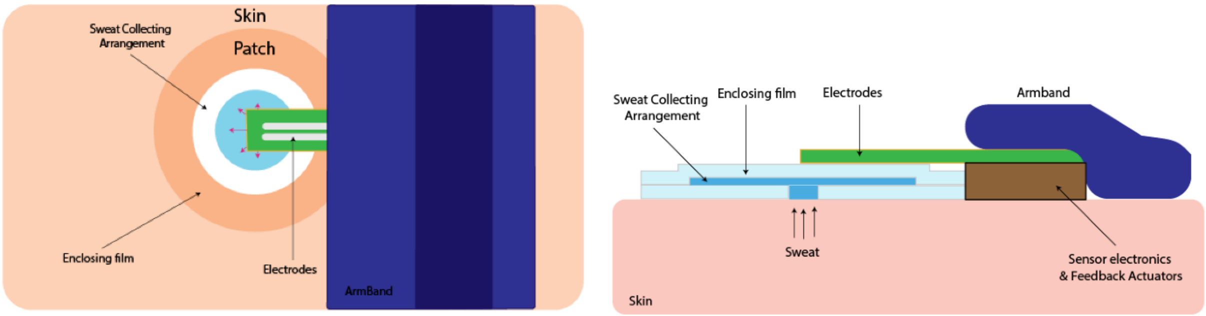

Figure S2: (a) Top view of the system showing patch, electrodes and arm band, (b) Side view of the system showing patch arrangement, electrodes, armband and electronic module.

This work presents a platform in a wearable form factor to measure the sweat rate and extract useful information from the data. Designing such platform requires an appropriate form of arrangement or platform to collect the sweat, measure its quantity while monitoring other parameters such as time and temperature; this information can then be used to produce insights about sweat rate, dehydration levels, fluid replacement recommendations, etc. Ideally, such system should also be able to provide the user with feedback of these parameters at desired intervals either visually or in other forms. The growth of consumer electronic devices including 'smart' devices such as smartphones, smartwatches, activity sensors, etc. provides another capability to take use of the already available mobile computing power by integrating this kind of sweat rate information with them. The thought process of the details of the design and what sort of arrangement and propertied can this system have is discussed in this chapter in thorough detail. A brief illustration of such a wearable concept is shown in Figure S1 and S2.

Consider a scenario like a marathon where there can be tens of thousands of athletes both amateur and professional, a low-cost platform like this can not only provide a way for each participant to plan their hydration strategies to improve performance but also aid the race

organizers and the paramedics to manage any medical emergencies, making sure that each of the tens of thousands of participants is safe. We have validated the design as well as choice of materials by doing extensive experiments with respect to varying sweating intensities (flow rates), thickness of the device as well as the porosity of the material of the device.

Although, the electronic module prototype developed herein is used for simple detection, but it can easily be extended in its applications by connecting it to smart phone or other smart devices as that would allow storing the information from the patch and device and doing better analyses using information from the grid (internet). By making it IOT device the applications can be limitless as then the data can be used to learn and better predict an individual's performance in varied conditions.

2. PATCH DESIGN ALGORITHM

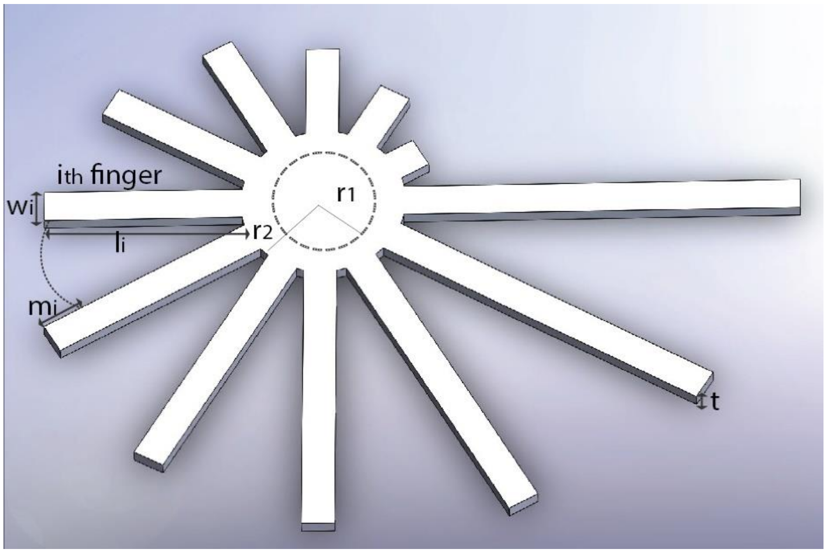

Figure S3: Parameters for design.

Table S1: Table for definition of parameters.

**Design Parameter**	**Definition**
$\boldsymbol{t}$	Substrate thickness
$\boldsymbol{r}, \boldsymbol{A}$	Radius, Area of the hypothetical fully circular patch
$\boldsymbol{r_1}, \boldsymbol{A_1}$	Radius, Area of port in membrane
$\boldsymbol{r_2}, \boldsymbol{A_2}$	$\boldsymbol{r_1}, \boldsymbol{A_1}$ + tolerance
$\boldsymbol{n}$	Number of fingers
$\boldsymbol{m_i}(\boldsymbol{l_{i+1}} - \boldsymbol{l_i})$	Difference b/w length of $\boldsymbol{i_{th}}$ & $(\boldsymbol{i}+\boldsymbol{1})_{th}$ finger
$\boldsymbol{w_i}$	Width of the $\boldsymbol{i_{th}}$ finger
$\boldsymbol{l_i}$	Length of the $\boldsymbol{i_{th}}$ finger
$\boldsymbol{g_i}$	Gap between the $\boldsymbol{i_{th}}$ & $(\boldsymbol{i}+\boldsymbol{1})_{th}$ finger
$\boldsymbol{SR}$	Sweating rate (biometrics)
$\boldsymbol{R}$	Desired readout resolution
$\boldsymbol{T}$	Time of application
$\boldsymbol{S}$	Desired size of the patch

Here we present, another way of looking at the calculations done in the main paper. All the geometric parameters of the patch must be decided based on the sweat rate per unit area (Q_s) of the person (which is based on sweat gland density in the region of application), desired readout resolution (R), and the desired size of the patch (S). However, these parameters are not all independent and have to follow geometrical constraints which are given by the following equations:

$$V_s = A_1 Q_s T = tA = V_a \tag{1}$$

where, V_s is the volume of sweat excreted by skin, A_1 is the area of the port in the membrane, V_a is the volume of sweat that can be absorbed by a paper having area A and thickness t.

To maintain a reasonable size for the patch size (easy to handle and applied in the desired area), a circular patch with radius r that can absorb the entire amount of sweat is assumed for an individual, using the patch for time T. The thickness is assumed as t. A thicker substrate estimate will be required for higher exercise rate to absorb a higher amount of sweat produced. Using Equation (1), the assumed r and the assumed t, we can find a value for A_1. For the sheet of paper to cover the entire port (sweat collection area) the following condition must be met:

$$A \geq A_1 > 1mm^2 \quad (2)$$

This equation assumes that 1 mm^2 is the size of an area of a skin having at least one sweat pore on the arm; typically, sweat pore size and spacing average 60–80 μm, and the density on the upper arm is about 100 glands per square centimeter[1]. If A_1 found from Equation (1) and the assumed values of A or r and t satisfy the above conditions, the assumptions can be used for the device design.

The number of fingers (N) in the patch and radial spacing between them (m_i), depend on the desired resolution of feedback (R), which in turn depends on the total time of application (T) and user preferences. In our design the sweat flows into the radial channels through the central region, the relation between these parameters

$$N = \frac{T - T_0}{R} \quad (3)$$

Where $T_0 = \frac{tA_1}{A_1 Q_s} = \frac{t}{Q_s}$ is the time taken to fill the central region i.e. ratio between the volume of the central region and the sweat flow rate. This T_0 can be chosen according to the user needs and reduces one degree of freedom in the model as it will fix the thickness t. However, if the size of the central region is small as compared to rest of the patch, we can ignore T_0 and take $N = \frac{T}{R}$ for rest of the calculations (simpler first order model), keeping A_1 to be equal to size of the port.

The minimum amount of spacing required between the fingers depends on the dye spot size according to visibility. The circular strip of area A is to be converted into the final desired shape with port area, A_1,

central circular region of the paper equal to port area, A_1, and the rest of the area, $A - A_1$ is distributed among the fingers.

$$A - A_1 = \sum w_i * l_i \quad (4)$$

where i represents the ith finger. Taking all w_i values to be the same and equal to w for maintaining uniform flow speed in the fingers as well as $m_i = l_{i+1} - l_i = m$ (for providing feedback to user at equal intervals of time when the width w of all fingers is assumed to be the same), the above equation simplifies to,

$$A - A_1 = w.[l_1 + l_2 + \cdots + l_n] = w.[l_1 + (l_1 + m) + (l_1 + 2.m) + \cdots + (l_1 + (N-1).m]$$
(5)

The only unknown in this equation is w. This obtained value of w is acceptable only if the constraint of the perimeter of port being greater than $\sum w_i$ across all fingers comes out to be true as in the Equation (5),

$$Nw < 2\pi r_1 \quad (6)$$

Together, Equations 5 and 6 define the design parameters for the sweat collection patch and enable mass customization of patches based on the required usage conditions. To determine the practicality of this design algorithm, the patch parameters required for measuring sweat rate were computed assuming three cases: an office person in a non- airconditioned environment using the patch for 30 minutes on the upper arm (with feedback at every 10 minutes), a competitive half marathon runner with patch on the dorsal hand targeting less than 90 minutes time to complete the race or ~6.5 minutes per mile pace), and a person exercising for 8 hours (such as in an ultramarathon) using the patch on his chest (with feedback after every 30 minutes). Here, the sweat rate values for all the three cases have been taken from Taylor et al.[1] Note that for cases 1 and 3, we use first order model whereas for case 2 use the second order model as an illustration. It is also important to note that even though we are starting with sweat rate value of an

average person to design the patch, based on the performance of the desired patch, the design can be optimized further by noting the actual sweat rate values during use.

2.1 Use Case Calculation and Implementation

To illustrate the algorithm and determine the practicality of this design, the patch parameters required for measuring sweat rate will be computed assuming three cases: a person at rest using the patch for 30 minutes and a person exercising for 8 hours (such as in a marathon) and a person with intermediate sweat rate using the patch for 90 minutes, with the values somewhere in middle of the other two cases. The sweat rate values for the three cases will been taken from literature (conditions defined by Taylor et. al. for skin area on upper arm).

2.1.1 Resting case

To show one case where there might be very less quantity of sweat generated and the time of application may also be low we consider a person in a resting state sweating at the rate of $0.25 \frac{mg}{cm^2.min}$ from the forearm (Taylor et. al.) wanting to monitor his sweat output for 30 min, if we follow the same algorithm defined in the theory section of the main paper, Equation (1) of main paper yields us the following:

$$V = A_1 * 0.25 \frac{mg}{cm^2.min} * 30\,min = t * A$$

As a first guess, let t = 100 µm and if we assume a hypothetical circular patch having radius r of 5 mm, we get from the above equation,

$r_1 = 5.78\,mm > r => A_1 < A$, i.e. condition is not satisfied. So, we can't use the above or a greater than above value of t. One can further check no matter what value of r we assume r_1 will

always come out to be greater than r which is not a possibility. So, let's iterate and use t = 50 μm and r = 5 mm again, the same analysis as described above will give us,

$$A_1 = \frac{50\,\pi}{3}\,m^2$$

$$r_1 = 4.08\,mm$$

$r_1 < r,\ 1mm^2 < A_1 < A,$ i.e. condition is satisfied. We can go ahead in the process further and look at a possible value of resolution desired. Since the time of application is 30 mins, let's say that the user desires to get the information every 10 min, this implies that the resolution, R is 10 min which further implies that the number of fingers, n is $\frac{30min}{10min}$ = 3. Assuming the dye spot size achieved manually to be m = 2 mm, we can start with the first finger length of 2 mm. That means the length of the fingers are going to be 2mm, 4mm and 6mm. For getting the final shape, we need w, so we use the Equation (3) from theory in the paper to get,

$$\pi((5 * (10)^{-3}\,)^2 - (4.18 * (10)^{-3}\,)^2) = w * (2 * (10)^{-3} + 4 * (10)^{-3} + 6 * (10)^{-3})$$

$$or\ w = 1.97\,mm$$

Using the check condition of Equation (4), we see,

$2\pi r_1 > (\sum w_i)\ or\ 2\pi(4.18) > 6.3!$, implying that the condition is satisfied. For ensuring proper sealing of the device to make sure that the sweat from the exposed area of the skin does not leak out of the device, we have added a tolerance value as small as possible by the fabrication method to the port hole (say, 0.1 mm) to get the central region of the patch giving it the dimension r_2 = r_1+tolerance = 4.18 mm.

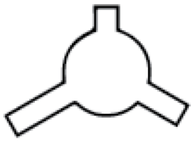

Figure S4: Geometric design for the resting case.

Figure S4, which illustrates the design and the patch that we obtain for time of application of 30 mins with desired resolution of 10 mins and user sweat rate of $0.25 \frac{mg}{cm^2 * min}$ has,

Thickness t, 50um,

Port radius r_1, 4.08 mm,

Central zone radius of the patch, r_2, 4.18 mm,

Width of the fingers w, 1.97 mm,

Number of fingers, n =3,

Difference between length of fingers m, 2mm

Circumferential gap between fingers g, 6.8 mm

And the length of fingers, 2mm, 4mm and 6 mm respectively.

2.1.2 Exercise case

Another case might be where there is high of amount of sweating rate and the time of application may also be very high, such as in a triathlon or an ultramarathon. We consider a

person in a heavy exercising state sweating at the rate of 0.9 $\frac{\text{mg}}{cm^2*\text{min}}$ from the chest[1] wanting to monitor his sweat output for 8 hrs. or 480 min, if we follow the same algorithm defined in theoretical section of the paper, Equation (1) yields us the following:

$$V=A_1*0.9\,\frac{\text{mg}}{cm^2*\text{min}}*480\text{ min} = t*A$$

As a first guess, let t = 200 um and if we assume a hypothetical circular patch having radius r of 10 mm, we get from the above equation,

r_1 = 1.86 mm < r, $1\,mm^2 < A_1 < A$, i.e. condition is satisfied. We can go ahead in the process further and look at a possible value of resolution desired. Since the time of application is 480 mins, and let's say that the user desires to get the information every 30 min, this implies that the resolution, R is 30 min which further implies that the number of fingers required, n is $\frac{480min}{30min}$ = 16. Assuming the dye spot size achieved manually to be m = 2 mm, we can start with the first finger length of 2 mm. That means the length of the fingers are going to be 2 mm, 4 mm, 6 mm, …, 32 mm. For getting the final shape, we need w, so we use the Equation (3) to get

$$\pi*((10*10^{-3})^2-(1.96*10^{-3})^2) = w*(2*10^{-3}+\cdots+32*10^{-3})$$

$$or\ w = 1.11\ mm$$

Using the check condition of eqn. 4, we see,

$2\,\pi\,r_1 > (\sum w_i)\ or\ 2\,\pi\,(1.86) > 16*1.11\ or\ 11.6 > 17.7!$, implying that the condition is not satisfied. Here we have added fabrication method limiting feature size (say, 0.1 mm) to the port radius to get the radius of the central zone of the patch Hence, we cannot use the parameters (t, r) as (200 µm, 10mm) and we must reiterate from the beginning.

Let (t, r) be (500 μm,10mm) instead and proceeding through the same steps one can see that with these values, r_1 = 3.4 mm and w = 1.0135 mm. In this case if we check the condition $2\pi r_1 > (\sum w_i)$ or $2\pi(3.4) > 16 * (1.01)$ or $21.6 > 16.16!$, the condition is satisfied, and we got a solution. For ensuring proper sealing of the device to make sure that the sweat from the exposed area of the skin does not leak the device, we have added a tolerance as small as possible defined by the limits of the manufacturing method used (say 0.1 mm) to the port hole dimension to get central region of the patch giving it the dimension $r_2 = r_1$+ tolerance = 3.5 mm.

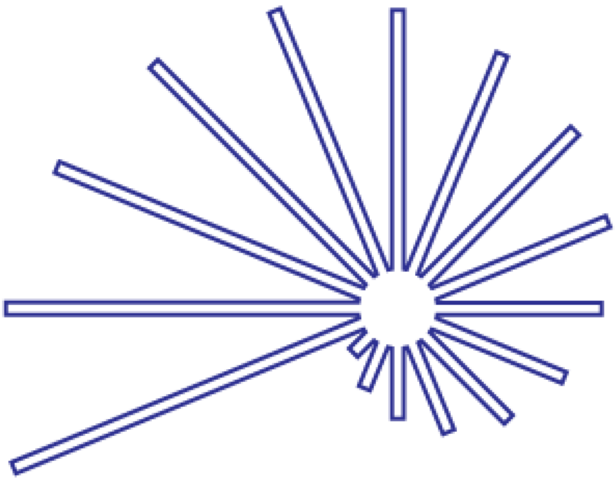

Figure S5: Geometric design for the exercise case.

Figure S5, which illustrates the design and the patch that we obtain for time of application of 480 mins with desired resolution of 30 mins and user sweat rate of 0.9 $\frac{\mathrm{mg}}{cm^2 * \mathrm{min}}$ has,

Thickness t, 500um,

Port radius r_1, 3.4 mm,

Central zone radius of the patch, r_2, 3.5 mm,

Width of the fingers w, 1.01 mm,

Number of fingers, n =16,

Difference between length of fingers, 2 mm,

Circumferential gap between fingers g, 0.33 mm,

And the length of fingers, 2 mm, 4 mm, 6 mm, …, 30 mm, 32 mm respectively.

2.1.3 Half marathon case

Let us try to design a patch using this algorithm for usage by an amateur athlete on the dorsal region of hand (back of the hand) who is targeting about 90 minutes to finish the race while practicing. For a person in a half marathon, the level of aerobic activity is high and can be considered in an exercise state. From [1], the sweat rate of the athlete in the dorsal hand region would be about 1.526 $\frac{\text{mg}}{cm^2*\text{min}}$. Let's design a patch which gives information to the athlete about his or her sweat loss after every 6.5 minutes beyond the first feedback point.

$$V=A_1*1.526\,\frac{\text{mg}}{cm^2*\text{min}}*90\text{ min} = t*A$$

As a first guess, let t = 180 µm (Paper thickness commonly available in labs) and after some trial and error if we choose a hypothetical circular patch having radius r of 11 mm, we get from the above equation,

r_1 = 4 mm > $r\ or\ 1mm^2 < A_1 < A$, i.e. the condition is satisfied. We can go ahead in the process further and look at a possible value of resolution desired. Since the time of application is 90 mins and the user desires to get the information every mile or every 7.5 min, this implies that for the first order model, the resolution, R is 7.5 min which further implies that the number of

fingers, n is $\frac{90min}{7.5\ min}$ = 12. The dye spot size that can be achieved comfortably by manual methods in lab is about m = 2 mm, we can start with the first finger length of 2 mm. That means the length if the fingers are going to be 2 mm, 4 mm, …., 24 mm. For getting the final shape, we need w, so we use the Equation (3) to get

$$\pi\ ((11 * 10^{-3})^2 - (4.5 * 10^{-3})^2) = w * (2 * 10^{-3} + \cdots + 24 * 10^{-3})$$

$$or\ w = 2.03\ mm$$

Using the check condition of Equation 4, we see,

$2\ \pi\ r_1 > (\sum w_i)\ or\ 2\pi(4.5) > 12 * (2.03)\ or\ 28.25 > 24.6!$ which implies that the condition is satisfied. If the same calculations are done using the second order model, where the time taken to fill the central region $T_0 = \frac{t}{Q_s}$, let's say is 12.5 minutes, it will reduce one degree of freedom in the system and the patch with same dimensions will give a resolution of 6.5 minutes and become more accurate. For ensuring proper sealing of the device to make sure that the sweat from the exposed area of the skin does not leak the device, we will add a tolerance value of 0.5 mm (limited by laser manufacturing method available in the lab) to the port hole to get the central region of the patch giving it the dimension r_2 = r_1+ tolerance = 4.5 mm.

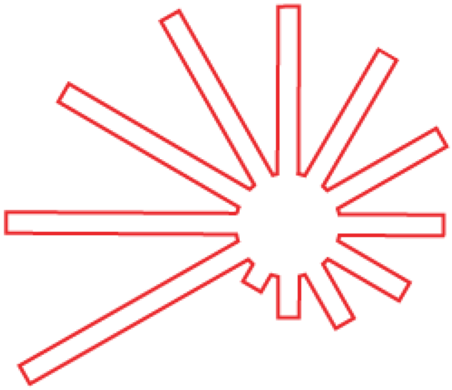

Figure S6: Geometric design for intermediate sweating case.

Figure S6, which illustrates the design and the patch that we obtain for time of application of 90 mins with desired resolution of 7.5 mins (first order model) or 12.5 mins + n(6.5 mins) (second order model) and user sweat rate of 1.526 $\frac{\text{mg}}{cm^2 * \text{min}}$ has,

Thickness t, 180um,

Port radius r_1, 4 mm,

Central zone radius of the patch, r_2, 4.5 mm,

Width of the fingers w, 2.03 mm,

Number of fingers, n =12,

Difference between length of fingers, 2mm

Circumferential gap between fingers g, 3.2 mm

And the length of fingers, 2mm, 4mm, …., 22 mm and 24mm respectively.

This design would be experimentally tested with a small modification of width taken to be 2 mm instead of 2.03 as that accuracy is not possible with the paper cutting method in lab. Similar approximations would be made to the sweating rate to the order which experimental equipment allows.

2.2 Feedback Module Circuit

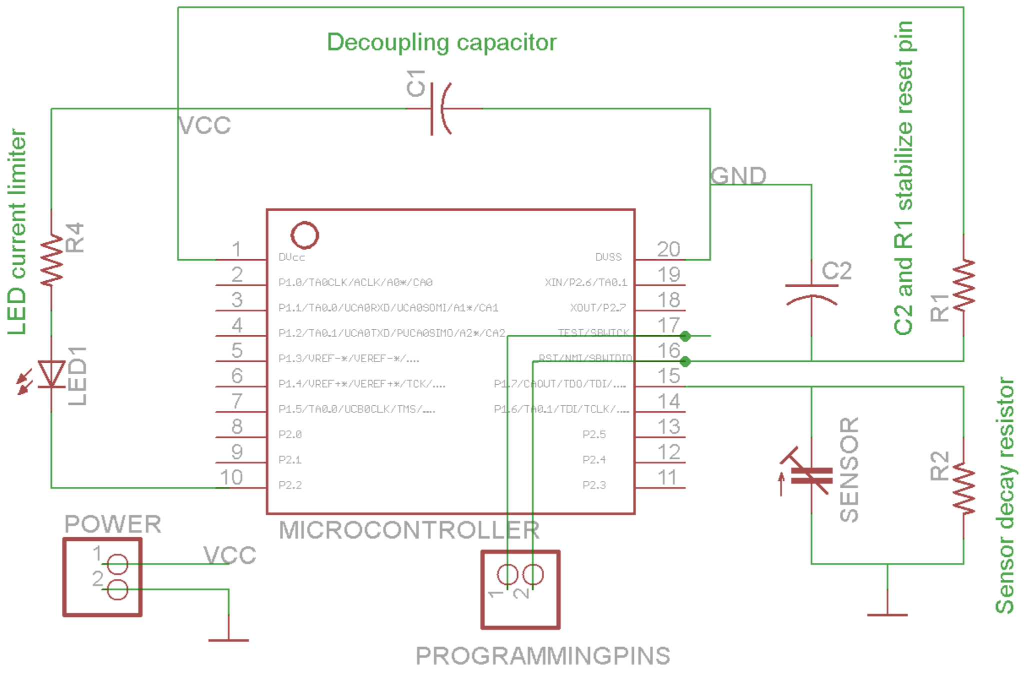

Figure S7: Capacitive based circuit design for the feedback module.

3. METHODOLOGY FOR WICKING MATERIAL SELECTION

Even though we have characterized the wicking performance of each substance, the wicking effect is also dependent upon the geometry of the patch. As a design of experiment strategy, to take that into account, without having to perform experiments for each design, we have come up with a methodology to estimate the time that would be taken by the patches of these three varied materials if they were experimentally tested in the form of geometries designed in section 2 using their primary wicking characteristics as tested by the experimental setup described in Figure S8.

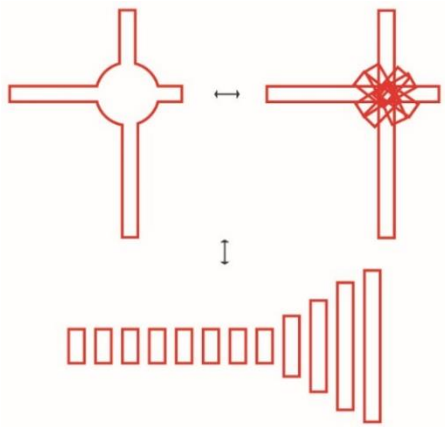

Figure S8: Assumption of the device as a collection of linear paper channels for calculation of the experimental wicking time.

The central circular portion of the design is approximated as a series of straight channels kept in a circular fashion with one end overlapping as shown in the figure S8. The number of channels have been estimated as $n=(2*\pi*r/w)$ and the channels corresponding to the fingers extend out from the circular region simulating the patch fingers. Using this analysis, for the two extreme cases that were designed in the theory section as well as case which will be

experimentally tested, we can find out the time range in which the designs would fill using the average wicking speed of the hygroscopic material used in the patch.

The experiments for wicking were conducted under infinite reservoir conditions (pure wicking) whereas in the device the phenomenon that occurs is forced imbibition (i.e., wicking along with a driving flow rate), Nevertheless, the experiment provide a first-order approximation of time ranges for a comparative analysis of material performance. Moreover, the wicking tests were conducted on linear channels only, whereas in the device we have both linear fingers and a circular central channel, which might cause wicking speed variations. To compensate for this in the analysis, the circular paper portion is approximated as a series of straight channels kept in a circular fashion with one end overlapping. The number of channels have been estimated as $n = \left(2 * pi * \frac{r}{w}\right)$ and the channels corresponding to the fingers extend out from the circular region simulating the patch fingers. Using this analysis, for the two extreme cases that we designed in the theory section and the average wicking speed of the three materials we can find the first order approximation of the time range in which the designs would saturate. Table S2 illustrates the comparison. The average wicking speed of each of the three materials cellulose, nitrocellulose and glass fiber was found to be 3 x 10^{-4} m/s, 8 x 10^{-5}m/s and 9 x 10^{-4}m/s respectively. We select filter paper because of its high strength, low cost, and intermediate wicking speed as compared to other two papers. Even though it has poor dye retention capabilities, the radial discrete fingered design of the patch mitigates the drawback. In the design of the device, due to the placement of the dyes at the tips of the fingers (discrete boundaries), the dye is limited to its bound area for the functional time of the device. (Also, mass diffusion rate of the dye << Flow convection)

Since we are using data from the wicking tests that have been done on linear channels only whereas in the device we have both linear fingers and a circular central channel too, there might be variations in the numbers we get from this method as compared to actual metrics. But it

can give us an approximation for order of magnitude analysis

Table S2: Experimental time taken by the device if it was made of each of the three materials for two extreme cases described in theory.

**Material**	**Resting Individual**	**Exercising individual**
Glass Fibre	1 min 30 sec	19.5 min
Cellulose	4 min	11.04 hrs
Nitrocellulose	20 min	50 hrs

4. COMPARISON OF HYGROSCOPIC MATERIALS

A suitable material for use as a wicking material in a sweat collection platform must be economical, flexible, strong, not brittle and hygroscopic. Three hygroscopic materials commonly available in research laboratories: Cellulose filter paper, Nitro Cellulose filter and Glass fiber filter paper were investigated for advantages and disadvantages.

4.1.1 Cellulose

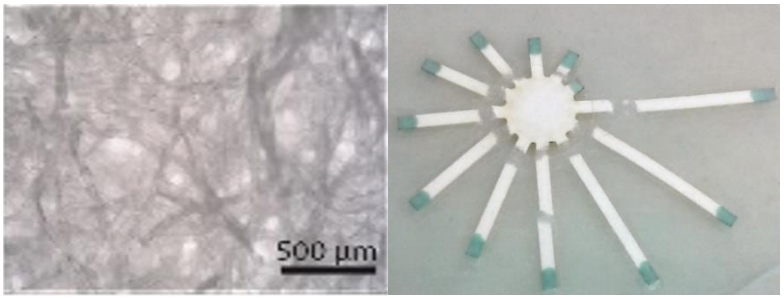

Figure S9: (a) Micrograph of cellulose paper showing the random arrangement of wood fibers, (b) Expected mechanical failure of the cellulose patch on bidirectional stretching.

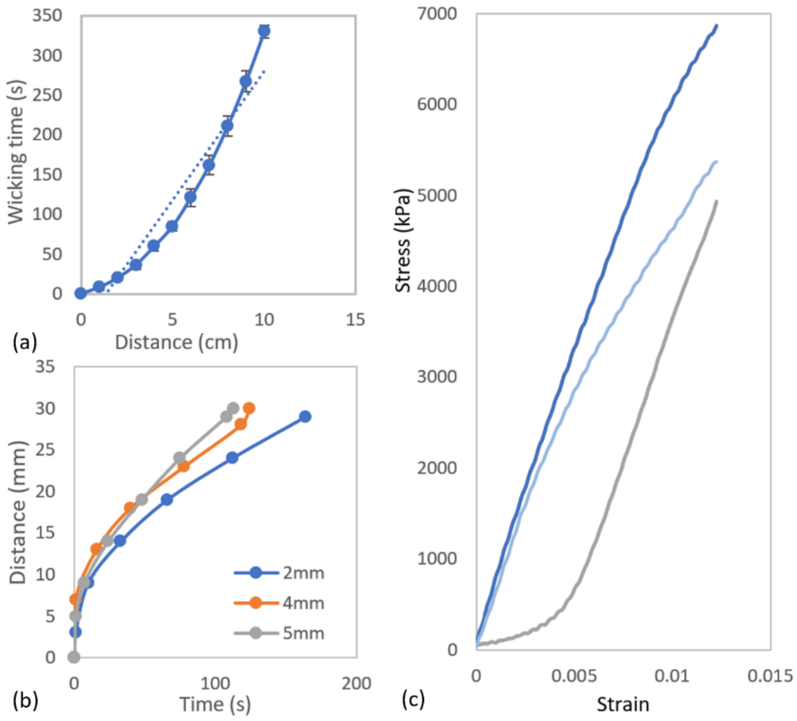

Figure S10: (a)Wicking speed of 2 mm width cellulose strip, (b) Comparative wicking speed curve for different cellulose strip widths, (c) Strength characterization of 2 mm cellulose paper strip through tensile testing (n=3).

Paper is inexpensive ($0.05 per patch) and is the strongest among the three tested materials, having an average Young's modulus of 472 MPa and yield strength of 7200 kPa. However, it is difficult to hold dyes on paper substrate due to its loose fibrous nature. The pore locations and sizes are not strictly controlled, and the material has average absorption (characterized by wicking speed) rate as compared to other two materials, which gives design flexibility.

4.1.2 Nitro cellulose

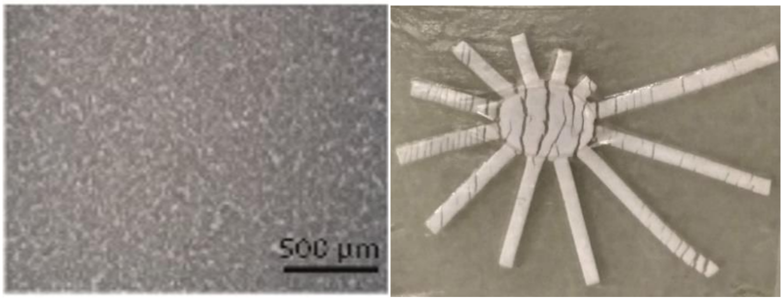

Figure S11: (a) Micrograph of nitrocellulose paper showing artificially engineered holes, (b) Unexpected uniform mechanical failure of the nitrocellulose patch on bidirectional stretching.

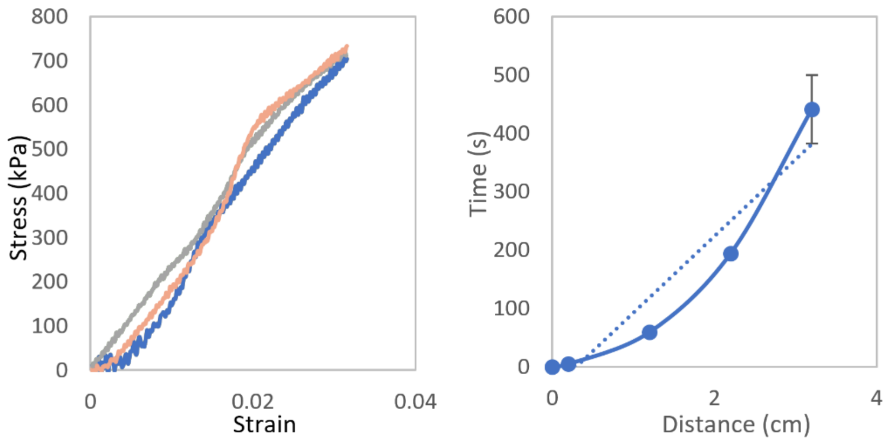

Figure S12: (a) Strength characterization of 2 mm nitrocellulose paper strip through tensile testing (n=3), (b) Wicking speed of 2 mm width nitrocellulose strip.

Nitrocellulose is an artificial membrane with precisely etched holes which provide it with a uniform morphology and higher dye holding capabilities. However, it is expensive ($1.5 per

patch), weak and brittle (Young's modulus of 25.4 MPa and yield strength of 760 kPa) and is difficult to handle. It has the lowest absorption rate (wicking speed) among the three materials.

4.1.3 Glass Fiber

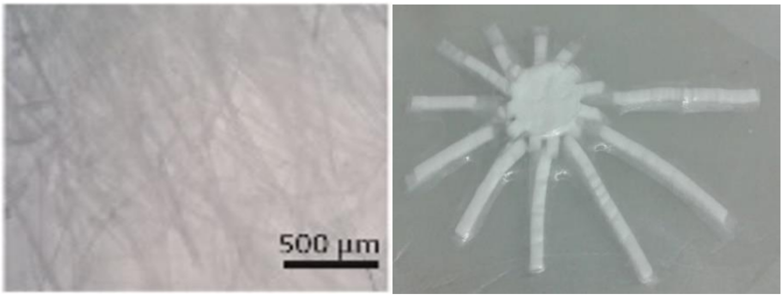

Figure S13: (a) Micrograph of glass fiber paper showing random arrangement of glass fibers, (b) Expected mechanical failure of the glass fiber patch on bidirectional stretching.

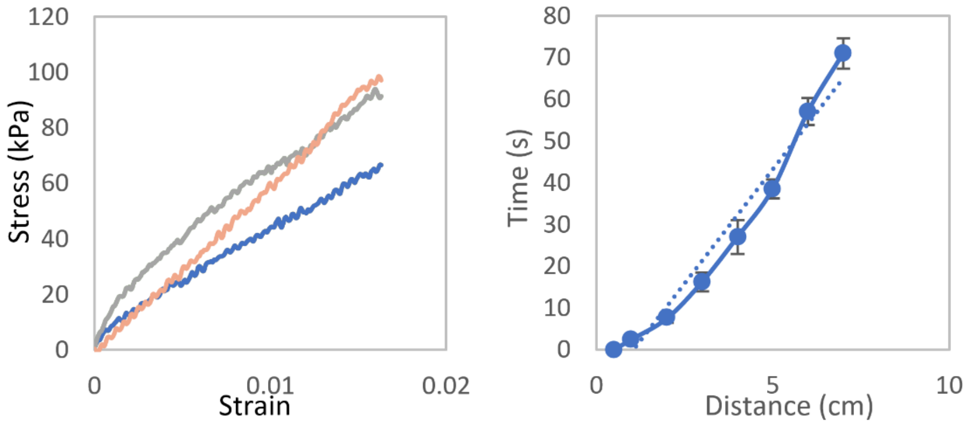

Figure S14: (a) Strength characterization of 2 mm glass fiber paper strip through tensile testing (n=3), (b) Wicking speed of 2 mm width glass fiber strip.

Glass fiber filter paper is expensive (3$ per patch), has low tensile strength (Young's modulus of 5MPa and Yield strength of 96 kPa) and turns out to be difficult to hold dyes since it's a collection of loosely assembled glass fibers. The pore size is not very strictly controlled (better than cellulose fibers) and the material has high absorption capacity (wicking speed) than other two materials.

4.1.4 Material summary

We select cellulose filter paper as the material of choice because of its high strength, low cost, and intermediate wicking speed as compared to other two papers. Even though it has poor dye retention capabilities, the radial discrete fingered design of the patch mitigates the drawback. In the design of the device, due to the placement of the dyes at the tips of the fingers (discrete boundaries), the dye is limited to its bound area for the functional time of the device. Also, the mass diffusion rate of the dye is many orders of magnitude less than the flow convection. There will be some negligible backflow, but that doesn't affect the user ability to have visual feedback as majority of the dye will remains at the tip due to greater number of boundaries resulting in lower surface energy and a stable state.

5. EXPERIMENTAL OBSERVATIONS AND EXPLANATIONS

Scaling analysis

To gain insight into device physics and understand the observations from the experiments we will analyze three microfluidic scaling numbers Capillary number, Reynold's number and Webber number. The capillary number expresses the ratio between the viscous and the capillary or surface tension or wicking forces and is written as (1)

$$Ca = \frac{\mu v}{\sigma}$$

where

μ is the coefficient of dynamic viscosity of the fluid

v is the velocity of the fluid

and, σ is the air-water surface tension.

The Reynolds number expresses the ratio between the inertial and viscous forces in the flow and can be written as (2)

$$Re = \frac{\rho v D}{\mu}$$

where

ρ is the density of the fluid,

v is the velocity of the fluid,

D is the hydraulic diameter [] or supp

μ is the coefficient of dynamic viscosity

The Webber number expresses the ratio between the inertial and the capillary forces and is written as (4)

$$We = \frac{\rho v^2 D}{\sigma}$$

Where

ρ is the density of the fluid,

v is the velocity of the fluid,

D is the hydraulic diameter [] or supp

and, σ is the air-water surface tension.

One can note that $Ca.Re = We$

The platform consists of two distinct regions; the central circular portion and the rectilinear strips. The flow coming from the source is going to be the same in the whole device, so (5)

$$v_c(2\pi rt) = q = 12v_s(wt)$$

Where v_c is the fluid velocity at a radial distance r from the center of the port (circular region) and v_s the fluid velocity in the strips (where flow rate $q^* = \frac{q}{12}$). In each of the two zones because of different velocity profiles and geometries (which affects hydraulic diameter), the numbers will play out differently. Hydraulic diameter is defined as 4A/P where A is the area of cross section of the flow an P is the perimeter of the wetted cross section. Using this definition,

the hydraulic diameter of the two regions in the device are found to be $2t$ and $2(\frac{1}{\frac{1}{t}+\frac{1}{w}})$ for the central and the strip region respectively. Where t is the thickness of the device and w is the width of the strip.

<u>Qualitative explanation:</u>

<u>Velocity</u>

When the flow starts in the device the velocity is infinite which can be seen from eqn 5 but rapidly decreases as the radius increases till the end of the circular region and then settle down to a constant value (with minor variations due to unequal length of the strips) in the strip.

This means that all the three scaling numbers (which are proportional to v, other parameters are constant values) begin with a very large value at r=0 and then settle down to a constant value.

<u>Spatial variation</u>

This implies that in the central region inertial forces are dominant over viscous forces which dominate over capillary forces in the beginning but at the end of the central region if one calculates these numbers, the order is rapidly seen to reverses as the flow spreads out and by the time the flow reaches the strips capillary forces dominate the other two. The flow changes from turbulent and unsteady all the way to creeping or Darcy flow regime (laminar & steady) when it reaches the strips and hence the farther one goes from the center the better observed will be the compliance between theoretical and practical values. This explains why the initial few strips have higher deviations from the ideal behavior observed Figure 6(b) and (d).

Flow rate variation

At a given location in the patch, as the flow rate increases these numbers increase, capillary forces decrease, and inertial increase leading to better observed compliance with theoretical characteristic curve as shown in Figure 6(b) (since characteristic equations are derived from volumetric conservation).

Thickness variation

On scaling with thickness, at a given location and a given flow rate, Re number doesn't change, Ca and We numbers change inversely proportional to the thickness, hence with increase in thickness the inertial flow forces increase and we see more variation from the linear curve is seen especially for the initial few strips as seen in Figure 6(d).

This is because initially all the numbers will always be of high magnitude (approaching ∞) causing the hydrodynamic property values to be unreliable as has been observed experimentally too (higher standard deviation value for the central circular strip experimental parameters T_0). Because of the inertia, some of that behavior might also extend to few initial strips as we see for Strips 1,2,3 (higher variation from linearity)

Quantitative explanation:

$We = \frac{\rho v^2 D}{\sigma}$, $Re = \frac{\rho v D}{\mu}$, $Ca = \frac{\mu v}{\sigma}$, $\mu = 8.9 * 10^{-4}$, $\sigma = 73 * 10^{-3}$, ρ=997

Zone 1 (central region)

$v = \frac{q}{(2\pi r t)}$,D = 4A/P= 2t,

At r->0

v->∞, Re->∞, We->∞, Ca->∞

At $r = 4\ mm$, flow rate of 0.05 ml/h and t = 180 μm,

$$v = 3.1 * 10^{-6} \frac{m}{s}$$

$$Ca = 3.6 \ * 10^{-8}$$

$$Re = 1.2 \ * 10^{-3}$$

$$We = 4.4 * 10^{-11}$$

Zone 2 (Strips)

$v_s = \frac{q}{12wt}$, D =4A/P =2$(\frac{1}{\frac{1}{t}+\frac{1}{w}})$ = 2t if t<<w

For flow rate of 0.05 ml/h, t=180 μm and w = 2 mm,

$$v = 3.2 \ * \ 10^{-6} \frac{m}{s}$$

$$Ca = 3.6 \ * \ 10^{-8}$$

$$Re = 1.1 \ * 10^{-3}$$

$$We = 4.1 \ * 10^{-11}$$

So even though the scaling numbers decrease rapidly within zone1, theoretically there should not be much variation in hydrodynamic parameters while transitioning from zone 1 to zone 2. Also since $Ca < 10^{-5}$, the wicking forces are dominating at this flow rate in the strips.

Now if the flow rate changes by some certain amount other parameters unchanged, in both the zones the velocity will scale linearly as well as the Ca and Re number, however the We number will change quadratically as its proportional to square of velocity. With increasing flow rates, the value of these numbers will increase showing decrease in the surface tension forces and hence we expect less deviation with respect to the ideal characteristic curve that has been derived with a geometric volumetric equation (mass conservation).

The changes with thickness of the device will not affect Re number in zone 1 as they are independent of t but the Ca number, We number and the velocity would change inversely proportional to the thickness. Even in the zone 2 till $t \ll w$ (which is in our device or for any practical device parameters) the same behavior will be observed which means that as the thickness increases the wicking behavior starts dominating and we see more variations from linear curve derived from mass conservation.

Peel test results

The adhesion strength of the patch to skin is an important parameter for any patch/bandage; therefore, we investigated the bond strength between the patch and skin via a 180-degree peel test using a tensile testing machine (UTM, ADMET Inc, eXpert 4000). A 5 cm × 7 cm patch was attached to the forearm and subsequently peeled off using the tensile testing machine at a rate of 5 cm/min, while the applied load and displacement were recorded over time. The experiment was conducted in triplicate.

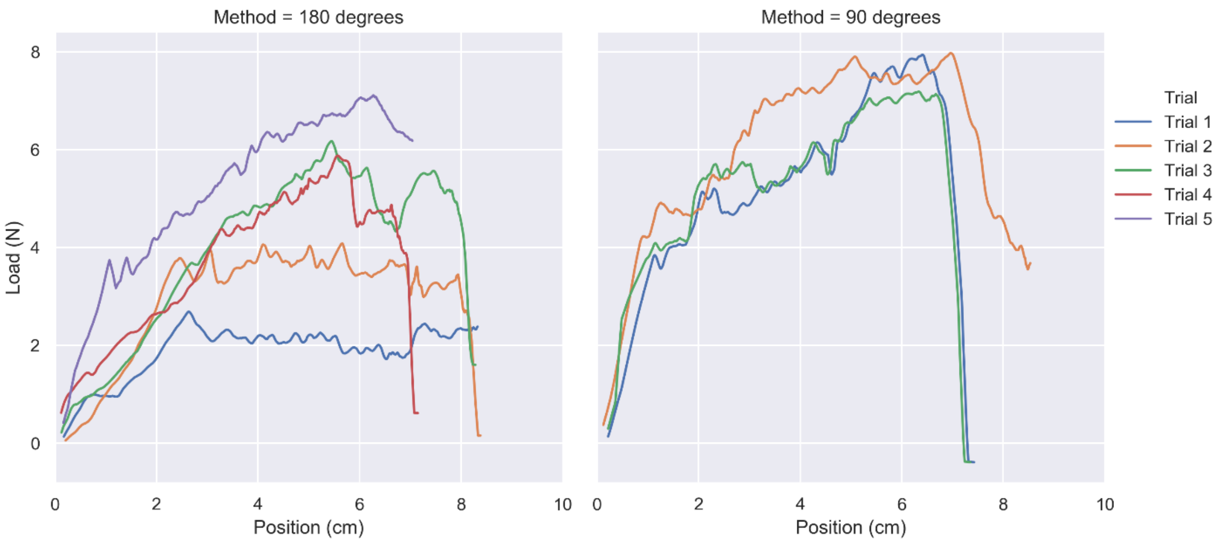

Figure S15: 90-degree and 180-degree bond strength test results; each series is a new patch (all 5 cm × 7 cm). The results of the peel test for three samples (each a different color) are plotted in the Figure S15 and show load variation due to variability of the skin (e.g., elasticity, hair density); nevertheless, the data reveal a bond strength in the range of 2−8 N for a peel distance of 5 cm (equivalent to 103−412 g/in), a range comparable to that of commercial bandages.[2]

References for Supplemental Material

[1] N. A. Taylor, C. A. Machado-Moreira, *Extrem. Physiol. Med.* **2013**, *2*, 4.

[2] S. Venkatraman, R. Gale, *Biomaterials* **1998**, *19*, 1119.
